# Spatiotemporal trends, evolving seasonality and symptoms of dengue in Bangladesh, 2019-2025

**DOI:** 10.1016/j.nmni.2026.101793

**Published:** 2026-06-11

**Authors:** Afsana Khan, Nazmul Sharif, Nadim Sharif, Shuvra Kanti Dey

**Affiliations:** a Department of Statistics, Jahangirnagar University, Savar, Dhaka, 1342, Bangladesh; b Department of Mathematics, Rajshahi University of Engineering & Technology, Rajshahi, Bangladesh; c Department of Microbiology, Jahangirnagar University, Savar, Dhaka, 1342, Bangladesh; d CMB Program, University of Vermont, Burlington, VT, USA

**Keywords:** Spatiotemporal distribution, Seasonality, Epidemiology, Dengue outbreaks, Bangladesh

## Abstract

**Background:**

Bangladesh has experienced an unprecedented increase in dengue incidence since 2019, with altered transmission patterns indicating changes in geographic spread, seasonality, and disease severity. We aimed to investigate ongoing trends in epidemiology, seasonality, and changes in disease prognosis from 2000 to 2025.

**Methods:**

We screened and analyzed data from the national dengue surveillance system (2000–2025) and environmental factors from the World Bank Climate Portal and national meteorological databases. We compared prevalence, incidence, rural to urban distribution, disease prognosis and seasonality between 2000–2018 and 2019–2025.

**Results:**

Dengue transmission in rural and semi-urban regions increased sharply from 2019, with rural-to-urban incidence ratios increasing from 0.11 to 0.41 (2000–2018) to 0.86–2.12 (2019–2025). SIRs declined in major hotspot cities despite significant increase of nationwide cases. After 2019, the seasonal peak shifted toward October–November, with significantly higher incidence (*p* < 0.05) from January–June. Associations between dengue incidence and temperature, humidity, and rainfall, slightly strengthened in the post-2019 period (*r* > 0.5). Available surveillance records indicated rapid clinical deterioration within 24–72 h following diagnosis among a subset of fatal cases.

**Conclusion:**

Our study suggests possible changes in epidemiologic and clinical trends of dengue and underscores the need for continuous vector surveillance, integrated virological and serological studies to address the emerging risks and predict the interventions.

## Highlights

• Dengue outbreaks in Bangladesh have shifted drastically since 2019, with rural areas now reporting equal to higher incidence than major cities (hotspots).
• The seasonal peak has sharply moved forward in the year and dengue cases have increased during January–June, traditionally found low-risk periods.
• Environmental factors, including temperature, humidity, and rainfall, are more strongly correlated with dengue incidence in recent years, suggesting a probable ecological adaptation of vectors.
• Fatal outcomes among patients without warning signs of dengue have risen sharply.
• Our study highlights the immediate necessity for year-round surveillance of dengue, vector monitoring, and integrated genomic and serological investigations.

## 1 Introduction

Dengue is a mosquito-borne acute febrile viral disease that has spread to more than 130 countries worldwide [1]. An estimated 400 million infections occur annually, posing a substantial public health threat, particularly in tropical and subtropical regions [1,2]. Approximately half of the global population is currently at risk of dengue infection. Despite its expanding geographic distribution and increasing disease burden, no universally effective antiviral therapy exists, and vaccine strategies remain limited in their population-wide applicability [1,2]. The burden of dengue is disproportionately higher in Asia, where favorable climatic conditions, rapid urbanization, and high population density facilitate sustained transmission.

Bangladesh, a densely populated country with a tropical monsoon climate, has been endemic for dengue outbreaks since 2000 [[3], [4], [5], [6]]. With a population exceeding 180 million, the country has experienced a substantial rise in clinically confirmed dengue cases, ranging from 100,000 to 400,000 annually in recent years [[7], [8], [9]]. Prior to 2019, reported annual hospitalizations rarely exceeded 100,000 cases [3]. Historically, dengue outbreaks were largely confined to major urban centers, particularly Dhaka, Chattogram, and Khulna, where unplanned urbanization and high population density created favorable conditions for vector proliferation. The density of *Aedes species* capable of transmitting dengue virus (DENV) typically increases during and after the monsoon season, contributing to seasonal spikes in incidence and mortality [[10], [11], [12]]. In addition, all four dengue virus serotypes (DENV-1 to DENV-4) have been reported in Bangladesh, with periodic shifts in dominant circulating serotypes. Previous studies have documented co-circulation of multiple serotypes, particularly DENV-2 and DENV-3, which have been associated with major outbreaks and severe disease manifestations. Changes in serotype predominance and secondary heterologous infections may contribute to altered epidemiological and clinical patterns through antibody-dependent enhancement.

Since the large-scale outbreak in 2019, however, the epidemiological landscape of dengue in Bangladesh has shifted markedly. Cases have surged beyond previous records, and transmission has expanded into semi-urban and rural regions across the country [7,13,14]. Increasing reports suggest that dengue is no longer predominantly an urban disease in Bangladesh. Although rural transmission has been extensively documented in other countries including India, Sri Lanka, Malaysia, and Colombia [[15], [16], [17], [18], [19]], systematic analyses quantifying the magnitude of rural expansion in Bangladesh remain limited. Concurrently, changes in the clinical presentation and progression of dengue have been increasingly reported following the 2019 outbreak [4,7].

Dengue infection manifests across a wide clinical spectrum. Approximately 50–60% of infections are asymptomatic, while symptomatic cases range from mild febrile illness to severe and potentially fatal disease [1,2,20,21]. According to the revised WHO classification, symptomatic cases are categorized as dengue without warning signs (DNWS), dengue with warning signs (DWWS), and severe dengue (SD) [1,2]. Patients with DWWS and SD require close monitoring and hospital management due to the risk of rapid deterioration [20]. Notably, recent national reports indicate an increasing proportion of fatalities among patients initially classified as DNWS, raising concerns regarding possible changes in disease progression, viral virulence, host susceptibility, or clinical recognition. However, comprehensive epidemiological, genotypic, and histopathological investigations addressing these fatal outcomes remain limited in Bangladesh.

The marked rise in case numbers, geographic expansion beyond traditional urban centers, altered seasonal distribution, and emerging changes in clinical severity collectively suggest a significant epidemiological transition in dengue transmission within Bangladesh [ [22], [23], [24]]. Despite growing evidence of these shifts, few studies have comprehensively examined the long-term spatiotemporal dynamics of dengue, the magnitude of urban-to-rural transmission changes, evolving seasonality following the 2019 outbreak, and alterations in clinical prognosis over an extended period.

In this study, we analyzed national surveillance data spanning 2000 to 2025 to characterize changes in dengue seasonality, geographic distribution, and clinical progression in Bangladesh. We specifically aimed to (i) quantify the shift in transmission from urban to rural regions, (ii) evaluate changes in seasonal patterns before and after 2019, and (iii) assess alterations in disease severity and fatality profiles, particularly among patients classified as DNWS. By providing a comprehensive longitudinal assessment, this study seeks to inform future vector surveillance strategies, public health policy, and clinical preparedness in response to the evolving dengue landscape in Bangladesh.

## 2 Materials and methods

### 2.1 Ethical approval

This study did not involve any primary data from human or animal participants. We obtained the ethical clearance from the Biosafety, Biosecurity & Ethical Committee (BBEC) at Jahangirnagar University and the approval number for this study is BBEC, JU/M 2025/02 (186).

### 2.2 Data and sources

We collected and screened data from the Dengue Dynamic Dashboard for Bangladesh from the health ministry and the national dengue surveillance system of the Directorate General of Health Services (DGHS, https://old.dghs.gov.bd/index.php/bd/home/5200-daily-dengue-status-report) [3]. The authors confirm that all of the used methods were performed in accordance with relevant and appropriate guidelines and regulations. The severity of dengue cases was defined based on the modified dengue severity classification by PAHO/WHO [1,2]. We collected environmental data, including average temperature of atmosphere, humidity, rainfall, UV index, and days of total rainfall in a month from the Climate Change Knowledge Portal from the World Bank Group (https://shorturl.at/mWdcp) and Kaggle for national climate data in Bangladesh. We followed some of our previously published articles to conduct this study.

### 2.3 Dengue case definition and confirmation

During the study period, dengue cases were reported through the national surveillance system of DGHS. Laboratory confirmation was performed according to national guidelines using a combination of NS1 antigen detection, anti-dengue IgM serology, and reverse transcription polymerase chain reaction (RT-PCR) when available. Though diagnostic capacity increased after the 2019 outbreak through expansion of testing facilities, increased clinical awareness, and enhanced reporting systems, surveillance coverage is still underdeveloped.

### 2.4 Statistical analysis

We used proportion, rate, and frequency for the categorical variables. Further, we used mean and standard deviation to find out the central tendency of continuous variables and normally distributed data. We calculated the incidence ratio and standardized incidence ratio (SIR) to find out the changes in distribution of dengue incidence in city areas and rural areas using equation i. The expected number of cases for the selected cities was determined by considering the total number of populations in the city areas as reference. We calculated the ratio of the month-wise incidence of dengue between city corporation areas and rural areas. Further, we used the Spearman's rank correlation analysis and Pearson correlation coefficient separately to determine the association between the environmental factors and incidence ratio of dengue. We used the Mann–Whitney *U* test to compare the monthly record of cases between 2000 to 2018 and 2019 to 2025. Additionally, we performed the Wilcoxon signed-rank test to compare the month-to-month changes of cases of dengue. Further, we conducted the Z-test, Chi-square test and *t*-test to compare the independent variables associated with disease prognosis and outcomes. P values ≤ 0.05 were considered statistically significant. We used the IBM SPSS Statistics for Windows (version 28.0; IBM Corp., Chicago, IL, USA) and Microsoft Excel 2023 for conducting the data screening and statistical analyses. We used QGIS (QGIS Development Team) to incorporate the map data.

i $$\text{SIR}=\frac{\text{Observed}\;\text{Number}\;\text{of}\;\;\text{Cases}}{\text{Expected}\;\text{Number}\;\text{of}\;\;\text{Cases}}$$

## 3 Results

### 3.1 Spatiotemporal distribution of dengue cases

**Fig. 1:**
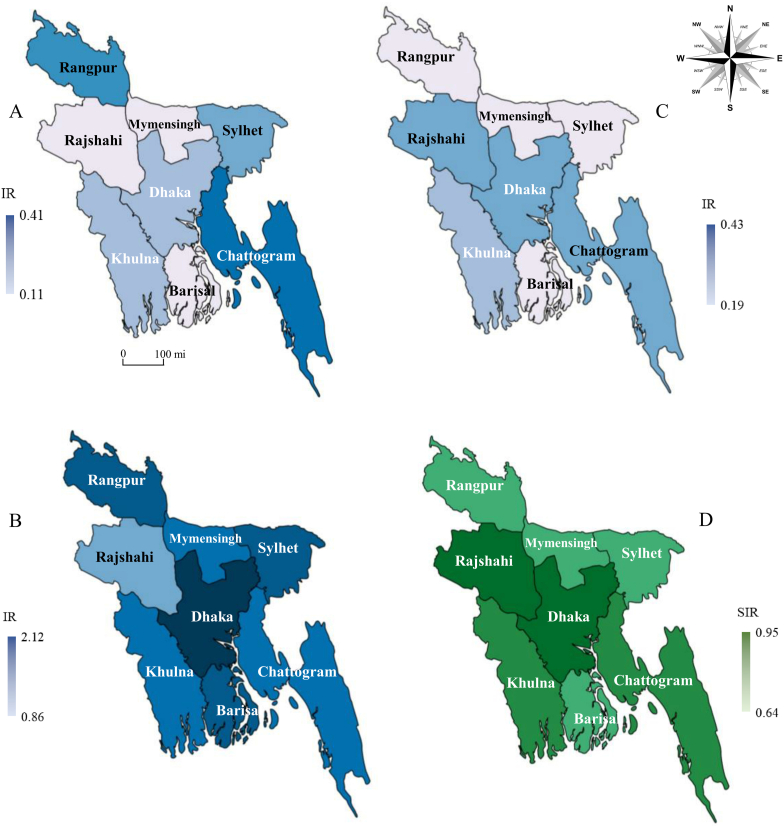
Spatiotemporal changes of incidence of dengue outbreaks in Bangladesh, 2000 to 2025. Incidence ratio of rural areas: city areas A. from 2000 to 2018; B. from 2019 to 2025; C. from 2000 to 2025 and D. standardized incidence ratio of city to rural areas from 2000 to 2025.

Dengue outbreaks are continuously reported in Bangladesh from 2000. The hospital reported cases remained below 15,000 and confined within the major city and urban regions including, Dhaka, Chattogram, Khulna, Sylhet and Rajshahi. However, during the 2019 outbreak and after in 2021, 2022, 2023, 2024, and 2025, confirmed cases passed 100,000 several times and cases were distributed throughout the country involving the cities and rural areas (Supplementary Table i and ii). We characterize this unusual distribution of cases by using the incidence ratio of rural areas to city areas involving the time frames from 2000 to 2025, from 2000 to 2018, and from 2019 to 2025 (Fig. 1). The ratio remained within 0.11 to 0.41 from 2000 to 2018 and within 0.19 to 0.43 from 2000 to 2025. This indicates that the overall incidence of dengue in cities is higher than in rural areas. Rural areas in Chattogram, Dhaka, and Rajshahi had comparatively higher incidence ratios than other regions. From 2019 to 2025, the incidence ratio ranged from 0.86 to 2.12 and the Central region (Dhaka), Northern regions (Rajshahi and Rangpur) and Southern regions (Barishal and Chattogram) showed significantly increased incidence in the rural areas (Fig. 1). Though the overall cases of dengue have increased from 10,000 in 2018 to 350,000 in 2023, the standardized incidence ratio reduced (SIR) in a range from 0.64 to 0.95 in the city areas. These differences in incidence ratios and SIRs in the city areas strongly indicate the spread of dengue into rural areas from 2019 to 2025.

### 3.2 Trends on seasonality of dengue outbreaks in Bangladesh

**Fig. 2:**
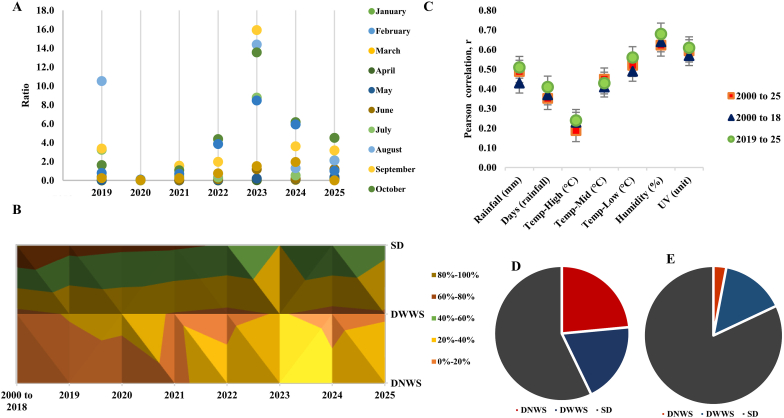
A. Month-wise ratio of prevalence of dengue during 2019-2025; B. Characterization of dengue symptoms severity among the cases during 2000-2025; C. Pearson correlation coefficient among the mean values of monthly environmental factors and dengue incidence; D. Proportionate distribution of severity of dengue cases during 2000-2018; and E. during 2019-2025.

Seasonality data on dengue had a distinct pattern over the last 26 years in Bangladesh. However, given the recent changes in massive spread of dengue across the country with record higher number of cases and fatalities, tracking the seasonality is essential. One distinct peak of cases and fatalities was confined within a period of six months starting from July to December and the top of the peak was contributed by August (160,000 cases), September (157,000 cases) and October (162,000 cases) (Supplementary Figure i). A sharp change in prevalence of dengue was observed between 2008 to 2018 and 2019 to 2025. From 2008 to 2018, we found that the month-wise order of confirmed cases as September > August > October > July > November, while from 2019 to 2025 it was October > August > September > November > July (Supplementary Table iii and iv). Further, we analyzed the month wise ratio of dengue cases from 2019 to 2025 (Supplementary Figure ii and iii). We found higher ratio of dengue cases in September 2023 (16.4) followed by August 2023 (15.1), October 2023 (14.3), and August 2019 (10.9), respectively (Fig. 2).

The month-wise changes of mean of cases from 2000 to 2018 and from 2019 to 2025 were significant (*p*-value <0.05) for compared twelve months. The most significant changes were observed in November, January, February, March, May and June (*p*-value 0.001) (Table 1). In month-to-month pair wise analysis we also found changes of cumulative monthly cases from one month to another month was statistically significant from 2000 to 2025 (Table 2). The highest significant difference of monthly cumulative cases was recorded in June to July (*p*-value <0.0001), and July to August (*p*-value <0.0001), indicating that the increase in cases was significantly different from previous month. On the other hand, the changes of monthly cumulative cases in August to September and September to October were not significant (*p*-value < 0.05) as this period indicated a steady highest peak in the morbidity curve (Table 2).

**Table 1:** Comparison of changes in dengue cases between 2008-2018 and 2019 to 2025 in Bangladesh. The Mann–Whitney *U* test was performed. Significant *p*-values (<0.05) indicated a reliable increase in cases.

Month	Mean (2000–18)	Mean (2019–25)	U Statistic	*p*-value
January	13.82	453.43	2	0.001
February	7.45	147.29	4	0.002
March	7.18	119.29	8	0.005
April	13.55	208.14	12	0.014
May	26.09	551.71	8	0.005
June	88.55	2231.14	7	0.005
July	290.18	11048.57	10	0.008
August	579.18	21845.14	11	0.011
September	688	21173.71	11	0.011
October	548.36	22409.29	6	0.002
November	245.18	14943.86	1	0.001
December	64.73	2693.5	10	0.022

**Table 2:** Pairwise comparison of dengue cases from 2000 to 2025. We used the Wilcoxon signed-rank test with significance level α = 0.05. A *p*-value of <0.05 indicated significant changes.

Month Pair	Mean_1_	Mean_2_	Test Statistic	p-value
**Jan vs Feb**	127	43.47	1	0.0044
**Feb vs Mar**	43.47	34	18.5	0.1971
**Mar vs Apr**	34	53.24	8.5	0.0292
**Apr vs May**	53.24	139.76	5	0.0046
**May vs Jun**	139.76	625.94	1.5	0.0009
**Jun vs Jul**	625.94	4108.71	0	<0.0001
**Jul vs Aug**	4108.71	8752.41	3	<0.0001
**Aug vs Sep**	8752.41	8230.47	45.5	0.1454
**Sep vs Oct**	8230.47	8257.47	53	0.2842
**Oct vs Nov**	8257.47	6010.24	9	0.0005
**Nov vs Dec**	6010.24	1441.76	0	0.001
**Dec vs Jan**	922.81	135.31	1	0.0019

Further, we conducted the Pearson correlation analysis to find the association of dengue incidence with different environmental factors for three different time frames, including 2000 to 2025, 2000 to 2018 and 2019 to 2025. The correlation of different environmental factors with the incidence of dengue was higher during 2019 to 2025 compared to other two-time frames (Fig. 2). Temperature, UV, humidity and rainfall were associated with the monthly incidence of dengue with a Pearson correlation coefficient above 0.5.

### 3.3 Evolving disease burden of dengue outbreaks

We used data of 11,230 cases from 2000 to 2018 and 45,421 cases from 2019 to 2025 to characterize the disease prognosis [3,4]. Fatality profiling showed that 82% of the death occurred among cases with severe dengue symptoms from 2000 to 2018, followed by 15% among DWWS, and 3% among DNWS (Fig. 2). On the contrary, a greater proportion of reported dengue-associated deaths occurred among cases initially classified as dengue without warning signs during the post-2019 period. However, individual patient-level clinical trajectories and timing of symptom progression were unavailable, limiting causal interpretation. Dengue-associated deaths ranged from 46% to 67% among cases with SD symptoms. The death patterns in 2023 and 2025 showed distinct characteristics with a death ratio of 1:1 among the DNWS and SV cases, which requires further investigation (Fig. 2).

**Fig. 3:**
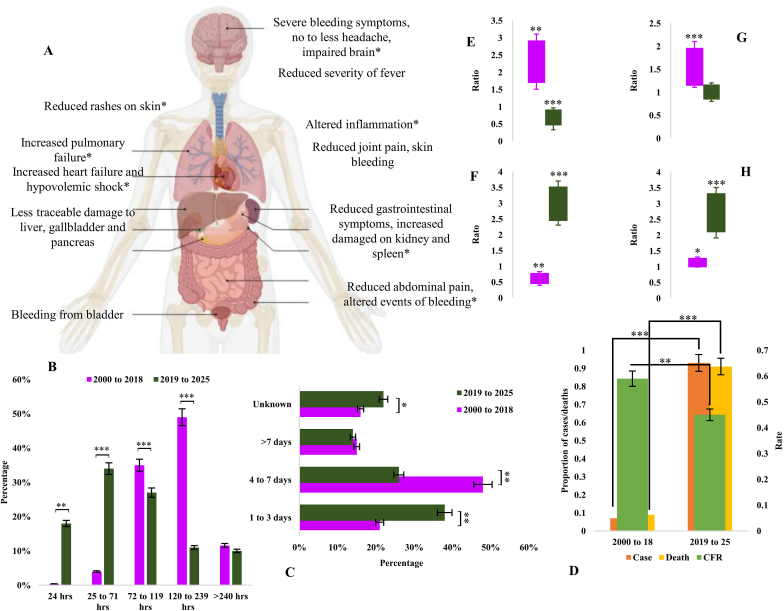
A. Comparative symptom profile of dengue cases between 2000-2018 and 2019-2025; B. Comparison of percentage of cases with fatality outcomes after having one or more symptoms of dengue; C. Comparison of percentage of cases with one or more severe symptoms after being positive for dengue; D. Comparison of proportion of dengue cases, fatalities and case fatality rate between 2000-2018 and 2019-2025; Comparison of ratios between 2000-2018 and 2019-2025 for E. Systemic symptoms; F. Lack of joint pain and skin rash; G. External bleeding; and H. Heart, brain, and lung impairment. We conducted the Mann-Whitney *U* test for non-normally distributed continuous symptom data, Chi square test of independence for the categorical symptom data, Z-test, and *t*-test where applicable. ∗ indicates ≤0.05, ∗∗ indicates ≤0.001, ∗∗∗ indicates ≤0.0001.

Symptom characteristics of dengue cases from 2019 to 2025 showed several changes compared to conventional dengue symptoms. Reduced proportion of high fever, rashes on skin, joint pain, abdominal pain, headache, and external bleeding were recorded among the confirmed cases. A significant proportion of reported deaths were associated with a sudden decline in platelet counts within 48 h of testing positive. Among other noticeable changes, increased proportion of pulmonary failure, hypovolemic shock, heart failure, kidney failure, impaired brain, bleeding in brain were significant among the death cases during 2019 to 2025 (Fig. 3). Sudden death with DNWS increased significantly. The frequency of fatality within 25 to 71 h of being positive was 39% followed by 72 to 119 h (28%) and 24 h (19%), respectively. The percentage of cases developing one or more severe symptoms within 1 to 3 days of being tested positive significantly increased from 20% to 39% (*p*-value 0.001) (Fig. 3). The case fatality rate reduced from 0.6 to 0.46 and proportion of cases and fatalities increased significantly from 0.04 to 0.96 (*p*-value 0.001) and from 0.08 to 0.92 (*p*-value 0.001) during 2019 to 2025.

## 4 Discussion

Dengue outbreaks have become a persistent and escalating public health challenge in tropical regions of South Asia, including Bangladesh [1,21,22]. In this study, we characterized recent shifts in the spatiotemporal distribution, seasonality, and clinical progression of dengue in Bangladesh, with particular emphasis on epidemiological changes observed from 2019 onward, a period overlapping with the COVID-19 pandemic. Our findings demonstrate three potential transitions: (i) an expansion of dengue transmission to semi-urban and rural areas, (ii) altered seasonal trends with prolonged transmission beyond the traditional monsoon period, and (iii) evolving clinical severity, particularly among patients previously categorized as dengue without warning signs (DNWS).

First, we observed a marked and uneven increase in dengue incidence in semi-urban and rural areas surrounding major metropolitan regions, including Dhaka, Rangpur, Sylhet, Khulna, and Chattogram after 2019. In several outbreaks between 2019 and 2025, rural-to-urban incidence ratios approached or exceeded 2:1, indicating that transmission intensity in some rural areas equaled or surpassed that of established city corporation hotspots. Compared to the pre-2019 period, many rural localities experienced a 300–500% increase in reported cases. These findings are consistent with recent national surveillance reports and regional studies documenting the geographic expansion of dengue beyond traditional urban centers [3,4,7,8,11,23]. Bangladesh has experienced rapid urbanization over the past two decades, accompanied by expansion of peri-urban settlements, increased population mobility, and changing water storage practices. These factors may facilitate dissemination of *Aedes* mosquitoes and dengue virus from metropolitan centers into surrounding semi-urban and rural districts [5,6,8]. Similar patterns have been reported in India, Sri Lanka, and Malaysia [15,16,18].

Two principal dengue vectors, *Aedes aegypti* and *Aedes albopictus*, are established in Bangladesh. *Aedes aegypti* predominates in densely populated urban settings, whereas *Aedes albopictus* is increasingly reported in peri-urban and rural environments. The expansion of these vectors into non-urban areas may partially explain the increasing incidence observed outside traditional dengue hotspots [9,15,17]. Increasing abundance and adaptation of *Aedes* spp. In peri-urban and rural environments, combined with co-circulation of multiple dengue virus (DENV) serotypes, likely contribute to sustained transmission. Recent entomological investigations have identified numerous breeding sites in newly affected areas, including construction sites retaining clean water and household water storage containers. Rapid population movement between urban hotspots and surrounding districts may have initially facilitated viral introduction; however, our findings suggest that local transmission now predominates in many rural settings. Together, these results indicate a potential expansion in dengue hotspots from historically urban centers to adjacent semi-urban and rural regions over the past seven years. These observations underscore the urgent need for integrated vector surveillance programs that comprehensively cover both urban and rural areas.

Second, although dengue transmission remains concentrated during the post-monsoon period, we identified alteration in seasonal patterns after 2019 outbreak. Historically, peak transmission occurred between August and October, with September typically representing the highest burden. In contrast, during 2019–2025, the peak shifted toward October, and transmission during January–June increased significantly compared to earlier years. This pattern suggests prolonged annual transmission and possible establishment of year-round viral circulation. Similar shifts in seasonal dynamics have been reported in Bangladesh and neighboring countries [4,7,22,23].

Our environmental analyses further support these observations. Associations between dengue incidence and climatic variables—particularly temperature, rainfall, humidity, and UV index—were stronger in the post-2019 period. Bangladesh experiences average annual temperatures ranging from approximately 19°C to 29°C, with substantial regional variation. These climatic conditions may increasingly favor vector survival, breeding, and viral replication across broader geographic and temporal ranges [[25], [26], [27]]. Notably, peak incidence and mortality consistently followed the monsoon season, reinforcing the established role of rainfall-driven vector proliferation. However, the strengthening correlations observed after 2019 may reflect ecological adaptation of vectors to wider environmental conditions, potentially enabling extended transmission periods. Collectively, these findings suggest that dengue seasonality in Bangladesh is becoming less sharply defined and more persistent throughout the year.

Third, we identified probable changes in disease severity and clinical progression after 2019 outbreaks. Most notably, the proportion of deaths occurring among patients classified as DNWS increased recently. Rapid declines in platelet counts, multi-organ dysfunction, and hypovolemic shock within 72 h of initial diagnosis were frequently observed among fatal cases, which requires urgent investigation. These findings suggest a possible shift in clinical phenotype and disease trajectory with larger outbreaks calling for detailed seroepidemiologic and clinical investigations.

Several mechanisms may explain these evolving clinical patterns. Reinfection with heterologous DENV serotypes is a plausible contributor, given documented co-circulation of DENV-2, DENV-3, and DENV-4 in major hotspots such as Dhaka and Chattogram [23,28,29]. Because a substantial proportion of dengue infections remain asymptomatic, some cases classified as primary infections may actually represent secondary or tertiary infections, which carry a greater risk of severe disease due to antibody-dependent enhancement. Additionally, post-COVID-19 immune modulation and underlying comorbidities might have influenced disease progression, as suggested by emerging literature on interactions between COVID-19 and other infectious diseases [30]. The potential introduction or evolution of distinct viral genotypes from neighboring regions might have contributed to altered pathogenicity and clinical outcomes. The observed changes in disease severity may partially reflect alterations in circulating serotypes and genotypes. Previous studies have reported co-circulation of DENV-2, DENV-3 and DENV-4 in Bangladesh. Because serotype-specific data were limited in the national surveillance database, the contribution of serotype shifts to the observed epidemiological changes could not be directly evaluated.

Despite its strengths, this study has several limitations. First, the absence of comprehensive vector surveillance data limited our ability to directly correlate entomological indices with spatiotemporal case distribution. Second, we did not include serotype or genotype data, which would have strengthened inferences regarding viral evolution and disease severity. Third, the analysis relied on national surveillance data, which may be influenced by reporting variability over time. Fourth, improvements in diagnostic capacity and surveillance reporting after 2019 might have contributed to increased case detection, which requires further investigation. Therefore, part of the observed rise in incidence might reflect enhanced surveillance rather than solely increased transmission. Fifth, nationwide environmental data may not fully capture local ecological heterogeneity associated with dengue transmission. Sixth, retrospective surveillance datasets lacked information on symptom onset, prior dengue infection history, comorbidities, and treatment interventions. Seventh, changes in surveillance intensity and diagnostic practices over time may have influenced temporal comparisons. Finally, the lack of individual-level immunological and serological data restricted our ability to distinguish between primary and secondary infections. Future studies incorporating prospective clinical cohorts, molecular characterization of circulating strains, and systematic vector monitoring will be essential to refine these findings.

## 5 Conclusion

In summary, our study provides evidence of a probable epidemiological transition in dengue transmission in Bangladesh since 2019. The disease has expanded geographically into rural areas, seasonal transmission patterns have shifted and prolonged, and clinical severity profiles have evolved, particularly among patients previously classified as having dengue without warning signs during these times. These findings highlight the need for strengthened surveillance systems, integrated vector control strategies, and comprehensive virological investigations to address the changing landscape of dengue in Bangladesh.

This is one of the first studies to report the changing seasonality, expansion of dengue with similar to higher incidence in the rural areas with repetitive outcomes over the last seven years, evolving disease progression profiles of dengue cases without warning symptoms and will provide initial baseline data to design vector surveillance, model seasonality and manage dengue cases. This study will aid in policy framework for real time monitoring of dengue outbreaks, adopting vector control strategies and clinical preparedness to tackle the severity of the outbreaks, including rural to urban areas.

## 6 Patient consent statement

Not applicable. This study did not involve any primary data from human or animal participants. We obtained the ethical clearance from the Biosafety, Biosecurity & Ethical Committee (BBEC) at Jahangirnagar University and the approval number for this study is BBEC, JU/M 2025/02 (186).

## Funding

This research was partially supported by Grants-in-Aid from the Ministry of Science and Technology, Government of the People's Republic of Bangladesh. The funder had no role in study design, data collection and analysis, decision to publish, or preparation of the manuscript.

## CRediT authorship contribution statement

**Afsana Khan:** Data curation, Formal analysis, Investigation, Methodology, Software, Writing – original draft, Writing – review & editing. **Nazmul Sharif:** Data curation, Formal analysis, Software, Writing – original draft, Writing – review & editing. **Nadim Sharif:** Conceptualization, Formal analysis, Software, Supervision, Validation, Visualization, Writing – original draft, Writing – review & editing. **Shuvra Kanti Dey:** Conceptualization, Software, Supervision, Validation, Writing – original draft, Writing – review & editing.

## Declaration of competing interest

The authors declare that they have no known competing financial interests or personal relationships that could have appeared to influence the work reported in this paper.

## Data Availability

All the necessary data have been made available in the Article and Supplementary appendix.
