# Supplementary material for: Spatiotemporal trends, evolving seasonality and symptoms of dengue in Bangladesh, 2019-2025
Source: New Microbes New Infect. 2026 Jun 11;72:101793. doi: 10.1016/j.nmni.2026.101793 (PMC13277491; doi:10.1016/j.nmni.2026.101793)
Supplement: Multimedia component 1 [file mmc1.docx]

Supplementary Figure i. Monthly distribution of dengue cases from 2008 to 2018.

Supplementary Figure ii. Monthly distribution of dengue cases from 2019 to 2025.

Supplementary Figure iii. Monthly distribution of dengue cases and fatalities from 2000 to 2025.

Supplementary Table i. Year-wise burden of dengue cases in Bangladesh.

| Month | 2008 | 2009 | 2010 | 2011 | 2012 | 2013 | 2014 | 2015 | 2016 | 2017 | 2018 | 2019 | 2020 | 2021 | 2022 | 2023 | 2024 | 2025 |
| --- | --- | --- | --- | --- | --- | --- | --- | --- | --- | --- | --- | --- | --- | --- | --- | --- | --- | --- |
|  |  |  |  |  |  |  |  |  |  |  |  |  |  |  |  |  |  |  |
| January | 0 | 0 | 0 | 0 | 0 | 6 | 15 | 0 | 13 | 92 | 26 | 38 | 199 | 32 | 126 | 566 | 1052 | 1161 |
| February | 0 | 0 | 0 | 0 | 0 | 7 | 7 | 0 | 3 | 58 | 7 | 18 | 45 | 9 | 20 | 166 | 399 | 374 |
| March | 0 | 0 | 0 | 0 | 0 | 3 | 2 | 2 | 17 | 36 | 19 | 17 | 27 | 13 | 20 | 111 | 311 | 336 |
| April | 0 | 0 | 0 | 0 | 0 | 3 | 0 | 6 | 38 | 73 | 29 | 58 | 25 | 3 | 23 | 143 | 504 | 701 |
| May | 0 | 1 | 0 | 0 | 0 | 12 | 8 | 10 | 70 | 134 | 52 | 193 | 10 | 43 | 163 | 1036 | 644 | 1773 |
| June | 0 | 0 | 0 | 61 | 10 | 50 | 9 | 28 | 254 | 267 | 295 | 1884 | 20 | 272 | 737 | 5956 | 798 | 5951 |
| July | 160 | 4 | 61 | 255 | 129 | 172 | 82 | 171 | 926 | 286 | 946 | 16,253 | 23 | 2286 | 1571 | 43854 | 2669 | 10684 |
| August | 473 | 125 | 183 | 691 | 122 | 339 | 80 | 765 | 1451 | 346 | 1796 | 52,636 | 68 | 7698 | 3521 | 71976 | 6521 | 10496 |
| September | 334 | 188 | 120 | 193 | 246 | 385 | 76 | 965 | 1544 | 430 | 3087 | 16,856 | 47 | 7841 | 9911 | 79598 | 18097 | 15866 |
| October | 184 | 154 | 45 | 114 | 107 | 501 | 63 | 869 | 1077 | 512 | 2406 | 8143 | 164 | 5458 | 21,932 | 67769 | 30879 | 22520 |
| November | 0 | 0 | 0 | 36 | 27 | 218 | 22 | 271 | 522 | 409 | 1192 | 4011 | 546 | 3567 | 19,334 | 42367 | 29652 | 5130 |
| December | 0 | 0 | 0 | 9 | 0 | 53 | 11 | 75 | 145 | 126 | 293 | 1247 | 231 | 1207 | 3731 | 7,637 | 9745 | 0 |
| Total | 1151 | 472 | 409 | 1359 | 641 | 1749 | 375 | 3162 | 6060 | 2769 | 10148 | 101354 | 1405 | 28429 | 61089 | 321,179 | 101271 | 74992 |

Supplementary Table ii. Year-wise cumulative cases, deaths and case fatality of dengue in Bangladesh from 2000 to 2025.

| Year | Cases | Death | CFR |
| --- | --- | --- | --- |
| 2000 | 5551 | 93 | 1.67 |
| 2001 | 2430 | 44 | 1.81 |
| 2002 | 6232 | 58 | 0.93 |
| 2003 | 486 | 10 | 2.05 |
| 2004 | 3434 | 14 | 0.4 |
| 2005 | 1048 | 4 | 0.38 |
| 2006 | 2200 | 11 | 0.5 |
| 2007 | 466 | 0 | 0 |
| 2008 | 1153 | 0 | 0 |
| 2009 | 474 | 0 | 0 |
| 2010 | 409 | 0 | 0 |
| 2011 | 1359 | 6 | 0.44 |
| 2012 | 671 | 1 | 0.15 |
| 2013 | 1749 | 2 | 0.11 |
| 2014 | 375 | 0 | 0 |
| 2015 | 3162 | 6 | 0.19 |
| 2016 | 6060 | 14 | 0.23 |
| 2017 | 2769 | 8 | 0.29 |
| 2018 | 10,148 | 26 | 0.26 |
| 2019 | 101,354 | 179 | 0.17 |
| 2020 | 1193 | 3 | 0.25 |
| 2021 | 28,429 | 105 | 0.37 |
| 2022 | 61,732 | 281 | 0.45 |
| 2023 | 321,179 | 1705 | 0.53 |
| 2024 | 101211 | 575 | 0.57 |
| 2025 | 74,922 | 302 | 0.40 |

Supplementary Table iii. Monthly average cases of dengue from 2008 to 2025.

| Month | Avg cases (2008–2025) | Avg cases (2008–2018) | Avg cases (2019–2025) |
| --- | --- | --- | --- |
| January | 185 | 14 | 453 |
| February | 62 | 8 | 147 |
| March | 51 | 7 | 119 |
| April | 89 | 14 | 208 |
| May | 231 | 26 | 552 |
| June | 922 | 89 | 2231 |
| July | 4474 | 290 | 11049 |
| August | 8849 | 579 | 21845 |
| September | 8655 | 688 | 21174 |
| October | 9050 | 548 | 22409 |
| November | 5961 | 245 | 14944 |
| December | 993 | 65 | 2694 |

Supplementary Table iv. Monthly average distribution of environmental factors from 2000 to 2025 in Bangladesh.

| Month | Avg. Tem (High in °C) | Avg. Tem (Mid °C) | Avg Tem (Low °C) | Rainfall (mm) | Days with rainfall | UV (unit) | Humidity (%) | Cases (number) |
| --- | --- | --- | --- | --- | --- | --- | --- | --- |
| January | 26 | 19 | 7 | 10 | 1 | 8 | 67 | 185 |
| February | 29 | 21 | 7 | 21 | 1 | 7 | 63 | 62 |
| March | 33 | 25 | 10 | 42 | 3 | 8 | 67 | 51 |
| April | 34 | 28 | 13 | 117 | 7 | 8 | 74 | 89 |
| May | 33 | 29 | 17 | 225 | 11 | 6 | 76 | 231 |
| June | 32 | 29 | 20 | 368 | 15 | 7 | 81 | 922 |
| July | 32 | 29 | 22 | 378 | 15 | 8 | 84 | 4474 |
| August | 32 | 29 | 22 | 388 | 15 | 9 | 83 | 8849 |
| September | 32 | 29 | 19 | 300 | 11 | 9 | 81 | 8655 |
| October | 32 | 28 | 15 | 190 | 6 | 8 | 78 | 9050 |
| November | 29 | 24 | 10 | 31 | 1 | 7 | 72 | 5961 |
| December | 26 | 19 | 8 | 6 | 0 | 7 | 70 | 993 |
